# Efficacy and safety of heat-sensitive moxibustion in the treatment of ulcerative colitis: A protocol for a systematic review and meta-analysis

**DOI:** 10.1097/MD.0000000000024078

**Published:** 2021-01-29

**Authors:** Jinlong Wang, Quanhui Zhang, Yongwen Deng, Gen Deng, Feihong Huang, Yiduo Zhou, Mingyan Jia, Haoran Yi

**Affiliations:** a College of clinical medicine, Jiangxi University of Traditional Chinese Medicine; b Affiliated Hospital of Jiangxi University of Traditional Chinese Medicine, Nanchang, China.

**Keywords:** heat-sensitive moxibustion, systematic review and meta Analysis, ulcerative colitis

## Abstract

**Background:**

Ulcerative colitis (UC) is a chronic nonspecific intestinal inflammatory disease with unclear etiology occurring in the colonic mucosa. Its clinical manifestations are characterized by recurrent abdominal pain, diarrhea, mucous pus, and blood stool. The severity of the disease varies, and itis characterized by a high recurrence rate. Because of its long course of disease, easy to relapse, protracted and difficult to recover, seriously affect the quality of life, increase the economic burden of patients and society, and even the risk of developing cancer, it has become one of the hot issues of general concern in the medical field. Heat-sensitive moxibustion therapy has shown strong advantages in the treatment of UC, and the curative effect is accurate. therefore, this paper will carry out a systematic evaluation and meta analysis of the efficacy and safety of heat-sensitive moxibustion in the treatment of UC.

**Methods:**

We will be searching 8 electronic databases, including PubMed, Embase, Web of Science, Cochrane Library, the China National Knowledge Infrastructure, Chinese Science and Technology Periodical Database, Wanfang Database, and Chinese Biomedical Literature Database. We will search above electronic databases from the beginning to December 2020, without any language restriction. Clinical efficacy, including total effective rate or cure rate, clinical symptom integral (abdominal pain, diarrhea, purulent stool), and recurrence rate will be accepted as the primary outcomes. The changes of cytokine Hs-CRP, IL-6, TNF-αlevels in serum, and improvement of colorectal mucosa will be used as secondary outcomes. RevMan 5.3 software will be used for statistical analysis. The result about the curative effect and safety of heat-sensitive moxibustion for UC will be presented as risk ratio for dichotomous data and mean differences with a 95% confidence interval for continuous data.

**Results:**

When this research program is completed, the relevant results can be obtained.

**Conclusions:**

The results of this study will provide reliable evidence for the efficacy and safety of heat-sensitive moxibustion in the treatment of UC.

**INPLASY Registration number:**

INPLASY20201101034.

## 1 Introduction

Ulcerative Colitis (UC) is a chronic nonspecific intestinal inflammatory disease with unclear etiology occurring in the colonic mucosa. Its clinical manifestations are characterized by recurrent abdominal pain, diarrhea, mucous pus, and blood stool.^[1]^ The severity of the disease varies, and it is characterized by a high recurrence rate. UC occurs most frequently in young adults.^[2]^ Epidemiological studies have shown that the incidence of inflammatory bowel disease is increasing year by year worldwide, and the prevalence of inflammatory bowel disease in China is about 11.6/100,000.^[3]^ At present, western medicine usually uses glucocorticoids, amino salicylic acid preparations, immunosuppressive agents, biological preparations, surgery, and other treatments for UC,^[4]^ but the clinical treatment effect is not good, with obvious side effects.^[5]^ Because of its long course of disease, easy to relapse, protracted and difficult to recover, seriously affect the quality of life, increase the economic burden of patients and society, and even the risk of developing cancer, it has become one of the hot issues of general concern in the medical field.^[6]^ Heat-sensitive moxibustion is a kind of traditional Chinese medicine external therapy, which has the characteristics of simple, safe, effective, and non-toxic side effects.^[7]^ In recent years, there have been more and more clinical reports on heat-sensitive moxibustion therapy for UC.^[8–11]^ However, there is still a lack of systematic evaluation on the efficacy and safety of heat-sensitive moxibustion therapy for UC in clinical practice. Therefore, the effectiveness and safety of heat-sensitive moxibustion in the treatment of UC will be systematically evaluated and meta-analyzed in this paper.

## 2 Methods

### 2.1 Study registration

This protocol was registered with the International Platform of Registered Systematic Review and Meta-Analysis Protocols (INPLASY) on 29 November 2020 and was last updated on 29 November 2020 (registration number INPLASY2020110134).

### 2.2 Inclusion criteria for study selection

#### 2.2.1 Types of studies

Clinical randomized controlled trials (RCTs) containing heat-sensitive moxibustion for UC were included, with no limitation of language and publication status.

#### 2.2.2 Types of participants

There are clear and recognized diagnostic criteria and efficacy criteria, and all patients are diagnosed as UC, regardless of gender, age, and origin of the case.

#### 2.2.3 Types of interventions

##### 2.2.3.1 Experimental interventions

Heat-sensitive moxibustion therapy, or mixed therapies based on heat-sensitive moxibustion will also be include.

##### 2.2.3.2 Control interventions

The control group will receive one of the following treatment methods: conventional pharma-cological therapy, no treatment, and placebo.

#### 2.2.4 Types of outcome measures

##### 2.2.4.1 Primary outcome

Clinical efficacy, including total effective rate or cure rate, clinical symptom integral (abdominal pain, diarrhea, purulent stool), and recurrence rate will be accepted as the primary outcomes.

##### 2.2.4.2 Secondary outcomes

The changes of cytokine Hs-CRP, IL-6, TNF-α levels in serum, and improvement of colorectal mucosa will be used as secondary outcomes.

### 2.3 Exclusion criteria

Non-RCTs; No exact diagnostic scale or therapeutic scale; No heat-sensitive moxibustion as the main treatment in the experimental group, and heat-sensitive moxibustion therapy was found in the control group. Repeated literature; theory and review literature; animal experiments; nursing research.

### 2.4 The retrieval methods and strategies of this study

#### 2.4.1 Electronic database retrieval

We will search 8 electronic databases, including PubMed, Embase, Web of Science, Cochrane Library, the China National Knowledge Infrastructure, Chinese Science and Technology Periodical Database, Wanfang Database, and Chinese Biomedical Literature Database. We will search above electronic databases from the beginning to December 2020, without any language restriction. And will searching the relevant literature by combining subject words with free words, search terms consist of disease (“Ulcerative Colitis” or “Idiopathic Proctocolitis” or “Colitis Gravis” or “Inflammatory Bowel Disease”) and intervention (“Heat-sensitive moxibustion” or “Thermal moxibustion” or “Thermosensitive hanging moxibustion” or “Heat-sensitizing acupoint moxibustion”) and research types (“randomized controlled trial” or “controlled clinical trial” or “random trials” or “RCT”). The PubMed search strategy is shown in Table 1.

**Table 1:** Retrieval strategies in PubMed.

ID	Query
#1	“Heat-sensitive moxibustion” [Mesh]
#2	(((Thermal moxibustion [title/abstract]) or (thermosensitive hanging moxibustion [title/abstract])) or (heat-sensitizing acupoint moxibustion [title/abstract])) or (heat sensitization [title/abstract])
#3	#1 or #2
#4	“Colitis, ulcerative” [MeSH]
#5	(((Idiopathic proctocolitis [title/abstract]) or (colitis gravis [title/abstract])) or (ulcerative colitis [title/abstract])) or (inflammatory bowel disease [title/abstract])
#6	#4 or #5
#7	(((Randomized controlled trial [title/abstract]) or (controlled clinical trial [title/abstract])) or (random trials [title/abstract])) or (RCT [title/abstract])
#8	#3 and #6 and #7

MeSH = medical subject headings, RCT = randomized controlled trials.

#### 2.4.2 Searching other resources

We will combine manual retrieval of literature resource database to search relevant conference papers that meet the inclusion criteria. In addition, the grey literature, as well as ongoing and recently completed studies, will be searched on Clinicaltrials.gov.

### 2.5 Data extraction and management

#### 2.5.1 Literature inclusion and data extraction

**Figure 1:**
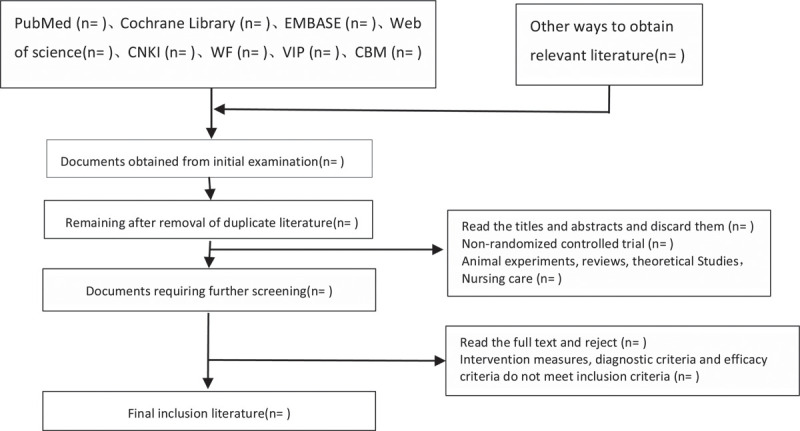
Flow chart of literature incorporation.

The 2 researchers independently read the title and abstract of the literature we obtained, read the full text of the trials that might meet the inclusion criteria to determine whether the inclusion criteria were truly met, and discussed the conflicting literatures or let the third researcher decide whether to include them. Two researchers independently extracted data from the included studies, including study design, intervention measures and methods, measurement indicators, results, methodological contents such as hidden grouping and blind method, etc, and a third evaluator checked the consistency of the data. If the required information is incomplete, we will contact the original author for the required data. The inclusion process of this study will be carried out as shown in Figure 1.

#### 2.5.2 Methodological quality evaluation

Two evaluators independently select the literature according to the inclusion and exclusion criteria and cross-check. In case of disagreement, a third evaluator will assist in the decision. The extracted data included the first author, year of publication, number of patients, age, gender, intervention measures, outcome indicators, etc. The Jadad scale to evaluate quality into literature, including: random sequence (right 2 points, 1 points not clear, inappropriate 0), distribution, hidden (right 2 points, 1 points not clear, inappropriate 0), blinded (right 2 points, 1 points not clear, inappropriate 0), lost to follow-up and exit (describe 1 points, not describe 0); 0 to 3 is classified as low quality and 4 to 7 as high quality.

### 2.6 Statistical analysis

#### 2.6.1 Quantitative data synthesis

Meta-analysis will be performed using Rev Man5.3.0 software. The odds ratio and its 95% confidence interval will be used as the counting data, while the weighted mean difference and its 95% CI will be used as the measurement data.

#### 2.6.2 Assessment of heterogeneity

The heterogeneity test will be carried out first among all studies, I^2^ test will be used. When *P* > .1 and I^2^ < 50%, the fixed effect model will be used; otherwise, the random effect model will be used. When the clinical heterogeneity between the 2 studies is large, only descriptive analysis will be performed.

#### 2.6.3 Publication bias

When the number of qualified RCTs is sufficient, we will use the inverted funnel Egger to test the potential publication bias.

#### 2.6.4 Subgroup analysis

If the necessary data are available, subgroup analysis will be carried out according to different factors based on the type of UC, treatment cycle, each moxibustion treatment time, and the type of intervention in the control group.

#### 2.6.5 Sensitivity analysis

The purpose of sensitivity analysis is to determine the sources and confounding factors of heterogeneity. If the trial data is sufficient, low or high quality studies will be excluded one by one for sensitivity analysis.

## 3 Discussion

UC can be classified as “dysentery”, “intestinal dysentery” and “blood stool” in traditional Chinese medicine. It is characterized by abdominal pain, diarrhea, mucus, and blood stool.^[1]^ Heat-sensitive moxibustion is the use of ignited moxa hanging moxibustion in the heat-sensitive acupoints, stimulate the transmission of qi to the disease, and apply individualized saturated desensitization moxibustion, heat-sensitive acupoints under the stimulation of moxa heat can appear heat permeation, heat expansion, heat transfer, local non-heat remote heat, surface non-heat deep heat, non-heat sense of 1, or more moxibustion feelings, the emergence of heat-sensitive moxibustion feelings is the key to clinical efficacy.^[12,13]^ A large number of clinical studies have found that heat-sensitive moxibustion therapy method has obvious advantages in treating or improving the clinical symptoms of UC patients.^[8–11]^ It can effectively alleviate the clinical symptoms of patients, alleviate systemic and local inflammation, improve the speed of mucosal healing, reduce the recurrence of disease, and improve the quality of life of patients.^[4]^ In recent years, because of its remarkable curative effect, this treatment method has been paid more and more attention in clinic, but we have not seen the systematic evaluation of the effectiveness and safety of heat-sensitive moxibustion in the treatment of UC. Therefore, it is necessary to systematically evaluate the treatment of UC by heat-sensitive moxibustion in this study, which can provide evidence-based medicine evidence for future clinical guidance of the treatment of UC by heat-sensitive moxibustion.

## Author contributions

**Data curation:** Jinlong Wang, Gen Deng.

**Formal analysis:** Feihong Huang, Yiduo Zhou.

**Investigation:** Yongwen Deng, Mingyan Jia.

**Methodology:** Yiduo Zhou, Haoran Yi.

**Project administration:** Quanhui Zhang,

**Software:** Feihong Huang, Mingyan Jia.

**Supervision:** Yiduo Zhou, Haoran Yi.

**Validation:** Yongwen Deng, Jinlong Wang.

**Visualization:** Yongwen Deng, Jinlong Wang.

**Writing – original draft:** Jinlong Wang, Quanhui Zhang.

**Writing – review & editing:** Jinlong Wang, Quanhui Zhang.

## Abbreviations

Abbreviations: RCTs = randomized controlled trials, UC = ulcerative colitis.

## References

[1] Zhu Yannan. Advances in clinical and experimental research on treatment of ulcerative colitis by external treatment of TCM. Clin Stud Tradit Chin Med 2019;11:146–8.

[2] Li Juan Wang Yan Zhao Qingxi. Effect of age on pathological location and degree of ulcerative colitis patients. J Med Coll Qingdao Univ 2015;51:486–7.

[3] Qin OY Xue LY. Inflammatory bowel disease in the 21st century in china: turning challenges into opportunities. J Dig Dis 2012;13:195–9. 22435503 10.1111/j.1751-2980.2012.00579.x

[4] Bi Tingting. Advances in clinical research of acupuncture and moxibustion in the treatment of ulcerative colitis. Chin Folk Ther 2019;27:105–7.

[5] Guo BJ Bian ZX Qiu HC. Biological and clinical implications of herbal medicine and natural products for the treatment of inflammatory bowel disease. Ann N Y Acad Sci 2017;1401:37–48. 28891095 10.1111/nyas.13414

[6] Nie Lin Zhang Lijiu. Advances in the treatment of ulcerative colitis with traditional Chinese medicine. Anhui Med 2019;23:860–2.

[7] Deng Gen Yi Baoxiu Zhang Juan. Clinical overview of heat-sensitive moxibustion on periarthritis of shoulder. Liaoning J Tradit Chin Med 2020;47:203–5.

[8] Lu Liqun. Clinical observation on the treatment of ulcerative colitis by supplementing spleen and spleen Xieyin Huo Shengyang decoction with heat-sensitive moxibustion. Sichuan Tradit Chin Med 2017;35:95–7.

[9] Fan Hongli Ke Binxia Lu Shuhong. Observation on heat-sensitive moxibustion and warm moxibustion in ulcerative colitis. Bright Chin Med 2016;31:1604–7.

[10] Fan Hongli Ke Binxia Lu Shuhong. Treatment of 30 cases of ulcerative colitis with thermosensitive moxibustion at special point. Jiangxi Tradit Chin Med 2013;44:46–9.

[11] Guo Xiang Guo Fei. Treatment of ulcerative colitis with moxibustion with heat-sensitive acupoints [J]. Chinese Folk Therapy 2010;18:12.

[12] Chen Rixin Chen Mingren Kang Mingfei. Paying attention to heat-sensitive moxibustion is the key to improve the curative effect of moxibustion. Acupunct Res 2010;35:311–4. 21090337

[13] Chen Rixin Kang Mingfei. A moxibustion is effective with gas. Zhongguo Zhen Jiu 2008;44–6. 18257189

